# Neuroanatomical domain of the foundational model of anatomy ontology

**DOI:** 10.1186/2041-1480-5-1

**Published:** 2014-01-08

**Authors:** B Nolan Nichols, Jose LV Mejino, Landon T Detwiler, Trond T Nilsen, Maryann E Martone, Jessica A Turner, Daniel L Rubin, James F Brinkley

**Affiliations:** 1 University of Washington, Seattle, WA, USA; 2 University of California San Diego, San Diego, CA, USA; 3 Mind Research Network, Albuquerque, NM, USA; 4 Stanford University, Stanford, CA, USA

**Keywords:** Data integration, Neuroanatomy, Neuroscience, Ontology, Brain atlas, Neuroinformatics, Information retrieval, mri

## Abstract

**Background:**

The diverse set of human brain structure and function analysis methods represents a difficult challenge for reconciling multiple views of neuroanatomical organization. While different views of organization are expected and valid, no widely adopted approach exists to harmonize different brain labeling protocols and terminologies. Our approach uses the natural organizing framework provided by anatomical structure to correlate terminologies commonly used in neuroimaging.

**Description:**

The Foundational Model of Anatomy (FMA) Ontology provides a semantic framework for representing the anatomical entities and relationships that constitute the phenotypic organization of the human body. In this paper we describe recent enhancements to the neuroanatomical content of the FMA that models cytoarchitectural and morphological regions of the cerebral cortex, as well as white matter structure and connectivity. This modeling effort is driven by the need to correlate and reconcile the terms used in neuroanatomical labeling protocols. By providing an ontological framework that harmonizes multiple views of neuroanatomical organization, the FMA provides developers with reusable and computable knowledge for a range of biomedical applications.

**Conclusions:**

A requirement for facilitating the integration of basic and clinical neuroscience data from diverse sources is a well-structured ontology that can incorporate, organize, and associate neuroanatomical data. We applied the ontological framework of the FMA to align the vocabularies used by several human brain atlases, and to encode emerging knowledge about structural connectivity in the brain. We highlighted several use cases of these extensions, including ontology reuse, neuroimaging data annotation, and organizing 3D brain models.

## Background

Large-scale human brain imaging initiatives are generating Big Data to characterize normal and neuropsychiatric brain structure and function. The Alzheimer’s Disease Neuroimaging Initiative (ADNI, [1]), Human Connectome Project (HCP, [2]), NKI-Rockland Sample [3], and others provide researchers with unprecedented access to massive amounts of shared neuroimaging data. While tools and methods are available to support the visualization [4-8], data management [9-13] and analysis [14-17] of shared or privately collected neuroimaging data, these tools use different approaches to define, segment, and label neuroanatomical structures.

One important component of research in this domain involves the development of digital brain atlases, which provide both a template brain and neuroanatomical labels in a standard coordinate system. Imaging data from individual participants are aligned to the template and brain region labels are propagated over to provide context to observed features in the data (e.g., location of activation foci). Atlases (i.e., the brain template and anatomical label pair) can be based on manual or automatically labeled brain regions that are derived using volume-based or surface-based methods. Each brain atlas develops, adopts, or refines an anatomical labeling protocol [18-24]. As a result, the labeling protocols used to define the boundaries of neuroanatomical regions can vary widely across brain atlases, so collections of anatomical entities from different labeling schemes do not always stand in a relation that allows a one-to-one mapping. Thus, data annotated with labels from different atlases are difficult to compare based on labels alone.

Previous efforts to reconcile terminologies in neuroimaging, referred to as the brain atlas concordance problem, have taken both quantitative and qualitative approaches. Taking a quantitative, bipartite graph approach, Bohland et al. demonstrated that different brain atlas labeling schemes lack a high degree of spatial concordance when comparing labels that seemingly refer to the same anatomical structure [25]. Qualitative approaches, such as our own, organize anatomical labels through synonymy, relations, and class hierarchies that provide practical utility (e.g., information retrieval and data integration) without resolving fine-grained spatial discrepancies. These approaches are complementary and both will be necessary in identifying a satisfactory solution to the brain atlas concordance problem.

To improve our symbolic model of the brain atlas concordance problem the labeling protocols from each brain atlas need to be made explicit. However, the labeling protocols that define anatomical structure boundaries in brain atlases are generally published in natural language (i.e., as a manuscript) and lack the term definitions and relationships provided by a machine-readable ontology framework. Neuroimaging data and information encoded by these terms cannot be accurately interpreted, compared, correlated and applied across different studies. A similar standardization issue faces the development of white matter connectivity atlases [26,27], in which our understanding of human brain connectivity is rapidly evolving. As new white matter analysis methods and labeling protocols are developed, a proliferation of terms to label newly identified structures in white matter atlases will likely occur.

A robust semantic framework is needed to explicitly represent the anatomical labels from different atlases using relationships that describe anatomical structure. Our goal is to provide such a framework for human neuroimaging that will facilitate the integration and harmonization of data registered to standard coordinate systems with labels for structures in human brain atlases.

### Approaches to labeling brain structures

We selected brain atlases that are widely used in the human brain mapping community and harmonized the terms used in each atlas’ labeling scheme with the anatomical structures modeled in the Foundational Model of Anatomy (FMA) Ontology. In this section, we provide a summary of brain labeling protocols that describe the anatomical knowledge and spatial relationships encoded in the Talairach Daemon, Desikan-Killiany (i.e., FreeSurfer), and Anatomical Automatic Labeling (AAL) atlases, as well as NeuroLex. We conclude with a proposal for harmonizing all atlas and NeuroLex terms with classes in the FMA ontology.

#### ***Talairach daemon labels***

The Talairach Daemon (TD) is an information system that provides a mapping between 3D coordinates (i.e., Talairach coordinates) and specific brain structure labels [28]. It is a digital representation of the original Talairach atlas [19] that is hierarchically organized into five levels:

1. Hemisphere

2. Lobe

3. Gyrus

4. Tissue type

5. Cell type

For example, the label “Right Cerebrum.Temporal Lobe.Inferior Temporal Gyrus.Gray Matter.Brodmann area 20” represents a number of 3D coordinates in the Brodmann area 20 cell-type level, the gray matter tissue-type level and so on. While this approach has been broadly applied in human brain mapping, there are limitations when normalizing patient MRI scans due to natural morphological differences between individuals.

#### ***Desikan-Killiany atlas***

The Desikan-Killiany (DK, [29]) atlas is a gyral, surface-based parcellation scheme for labeling anatomical MRI scans. The anatomical labeling protocol was manually applied to 40 MRI scans to build a template brain with labels for 34 cortical regions of interest (ROI) per hemisphere. This atlas is packaged with the FreeSurfer MRI data analysis package [30] that provides researchers with access to a variety of image processing tools that includes labeling anatomical ROIs with a predefined set of terms.

#### ***Automated anatomical labeling***

The Automated Anatomical Labeling (AAL) brain atlas provides labels for 90 anatomical regions of interest (45 per hemisphere) from a single participant using magnetic resonance imaging (MRI) [20]. Anatomical structures (45 per hemisphere) were identified in a high-resolution MRI by manually tracing structures in each slice of a 3D volume. The AAL Toolbox for the Statistical Parametric Mapping Matlab package [17,31] provides researchers with a method for labeling brain regions in their data using the AAL protocol and corresponding vocabulary.

#### ***NIFSTD and NeuroLex***

The Neuroscience Information Framework (NIF) standard ontologies (NIFSTD) are developed to provide a consistent source of terminology for neuroscience concepts [32]. NIFSTD is not a brain labeling protocol nor is it tied to a particular spatial arrangement of brain regions, but is a collection of brain region labels and inter-relationships. Neurolex also represents a general mammalian hierarchy of brain parts, with each structure assigned a taxon rank at which it is generally considered to hold, whereas other ontologies, like the FMA, are more species specific. NIFSTD is a formal ontology constructed through the import of community ontologies with specific extensions for neuroscience, covering the major domains of neuroscience [32,33]. For community contributions, NIF maintains the Neurolex lexicon, where each entity within the ontology is exposed as a wiki page (http://neurolex.org), built using the Semantic Media Wiki Platform. Entities migrate from Neurolex into the more formal NIFSTD ontologies [33].

An important feature of the project is to clearly and explicitly define all of the terms that are used to describe data (e.g., anatomical terms, techniques, organism names). The NIF gross anatomy module was largely based on the NeuroNames hierarchy [34-36], re-coded in the Web Ontology Language (OWL), but has been extensively modified through contributions to Neurolex. Neurolex serves as a community platform where those with minimal knowledge of building ontologies can still contribute their expertise. Through programs such as the Neuron Registry project of the International Neuroinformatics Coordinating Facility (http://incf.org), Neurolex is growing into a significant knowledge base for neuroscience. However, NeuroLex does not currently provide the framework necessary to correlate the terms from different brain labeling schemes.

### Our approach: the Foundational Model of Anatomy ontology

The Foundational Model of Anatomy Ontology (FMA) [37] is an open source reference ontology for the domain of anatomy that takes into account, at all biologically salient levels of organization, the entities and their spatio-structural relations which constitute and form the structural phenotype of vertebrates with a special emphasis on the human organism. It is based on a unifying theory that explicitly defines anatomy and its content from the structural point of view. In particular, it provides a framework that can incorporate and accommodate all entities under the purview of the anatomy domain.

The FMA is implemented as a computable information artifact and is primarily intended for developers of terminologies and application ontologies [38] in clinical medicine and biomedical research that require anatomical knowledge. Ontologists primarily value its merits because it is both broader and more fine-grained than extant anatomy texts or terminologies. For example, the FMA models both abstract, high-level concepts and leaf-level, fine grained concepts such as “Material anatomical entity” and “Brodmann area 1 of left postcentral gyrus”, respectively. This approach is not entirely consistent with the tradition-based representation of anatomy that clinical practitioners and biomedical researchers are taught in their training. Therefore, the benefits the FMA offers to end users are best realized through derived application ontologies [38,39] and biomedical software that utilize anatomical knowledge.

The principled framework provided by the FMA is flexible enough to capture the intended semantics of terminologies developed for more specific purposes. For example, application ontologies derived from the FMA (e.g., RadLex [40,41]) can be used to reconcile prevalent views of anatomy (e.g., radiologists or anatomy teachers) with an ontological representation of biological structure. Thus, knowledge extracted from the FMA can be abstracted to a level that is familiar to individuals in a given domain. The FMA can also incorporate annotations on anatomical entities that provide a mapping to external knowledge sources (e.g., ontologies or brain atlas terms), as well as a means to correlate between mapped terms. A central goal of this paper is to demonstrate how the FMA can be used to harmonize the growing number of neuroscience terminologies and provide a framework and use cases for developing useful biomedical applications.

### Construction and content

#### ***Authoring environment***

The FMA information artifact is implemented in Frames using Protégé, an authoring and editing environment created by members of the Stanford Biomedical Informatics Group [42]. Currently, the master copy of the FMA is stored in a relational MySQL database; however, many major biomedical ontologies (e.g., those in the OBO Foundry [43]) are now developed using OWL. OWL is now the standard language for describing ontologies on the Web, and there are ongoing efforts to translate the FMA into OWL. Previous attempts succeeded in creating a version of the FMA in OWL Full [44], and more recently a subset of the FMA was converted into OWL 2 [45]. The migration of the entire FMA into OWL 2 would greatly facilitate integration and interoperability with external ontologies and Semantic Web-related technologies. A strategy for this conversion is in early development, thus outside the scope of this paper; however, even without this migration, the current Frames version allows us to reconcile existing neuroanatomy terminologies.

### Enhancing neuroanatomy content in the FMA

The neuroanatomical content of the FMA was enhanced with detailed modeling for cerebral hemisphere brain labeling schemes, cerebral sulci, white matter structures, and neural connectivity relationships. These enhancements were designed to support use cases in human brain imaging by incorporating four major terminologies, described above, that are widely used for annotating neuro-related data (i.e., Talairach, Desikan-Killiany, AAL, NeuroLex). Our goal was to augment the FMA with the spatio-structural properties needed to represent different brain labeling schemes, while maintaining a single coherent framework. By accommodating different views within the same framework, we can use the enhanced FMA properties to correlate disparate brain labeling schemes. In the next section we describe the extension in more detail. Note that in this paper we represent FMA classes in Courier New font and relationships in ***bold italic***.

#### ***Cerebral hemisphere labeling***

For a given anatomical labeling protocol, the terms used to label or annotate brain structures may refer to neuroanatomical entities at different levels of granularity or using disparate features (e.g., morphological vs. cytoarchitectural) to define the boundaries of specific structures and their corresponding labels. This means that there may not be a direct or one-to-one correspondence between the terms from different atlases or terminologies. However, by mapping these terms to the FMA, the ontological structure of the FMA explicitly defines what entities are represented by the terms and how they correlate with one another according to the properties and spatio-structural relationships established for them in the FMA. In this section we provide a technical overview of how the FMA was enhanced to accommodate and correlate different brain labeling protocols.

**Figure 1:**
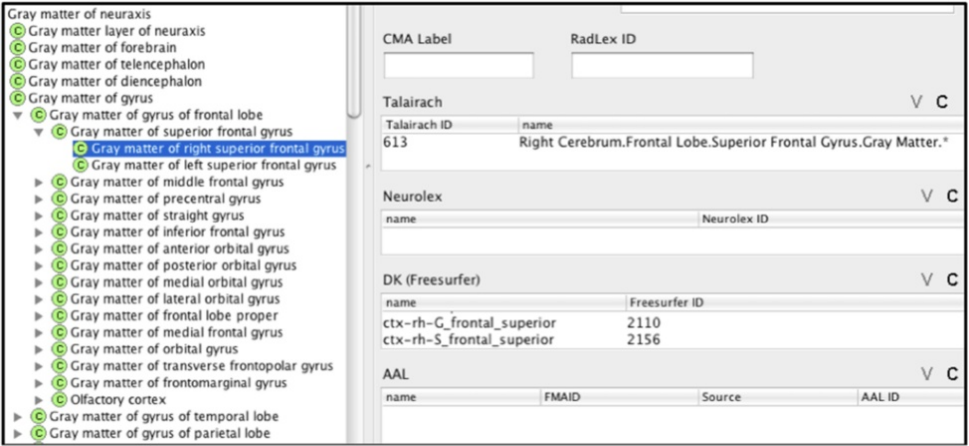
**Protégé screen capture showing the slots for the different terminologies.** In the example, the FMA class Gray matter of right superior frontal gyrus maps to Talairach and DK (Freesurfer).

To provide a mapping between different terminologies we used Protégé to introduce property slots (e.g., source names and unique identifiers) that link FMA classes to corresponding annotation terms (Figure 1). The labels used in each labeling scheme were manually correlated with a corresponding FMA term. A list of potential mappings was semi-automatically generated using direct string matching, synonymy mapping, lexical mapping, or by interpreting the symbols and abbreviations used in a given labeling protocol (e.g. “R” for “Right”, “ctx” for “cortex”, etc.).

#### ***Mapping NeuroLex***

Using the term matching approach described above, the terms from NeuroLex were mapped to a subset of classes from the FMA that model neuroanatomical knowledge. For example, Right frontal lobe is a direct string match between FMA and NeuroLex, Inferior horn of the lateral ventricle in NeuroLex is synonymous with *Temporal horn of lateral ventricle* in the FMA, and Lateral occipital cortex in NeuroLex is a lexical match to *Cortex of lateral occipital gyrus* in the FMA. The mappings were then manually incorporated into the FMA by adding specific NeuroLex identifiers to the newly defined NeuroLex_ID property.

#### ***Mapping Talairach***

Talairach Daemon annotations explicitly represent five levels of partonomy where the level of granularity for each neuroanatomical entity is denoted by a period. For example, the Talairach label Right Cerebrum.Frontal Lobe.Superior Frontal Gyrus.Gray Matter.Brodmann area 6 indicates a set of coordinates located on Right Brodmann area 6, on the Right superior frontal gyrus of the Right frontal lobe in the Right cerebral hemisphere. The actual neuroanatomical entity being represented here is “Brodmann area 6 of right superior frontal gyrus”, which exists in the FMA and is therefore directly mapped to the corresponding Talairach term.

Where appropriate and necessary in the ontology, we added new classes, properties and relations to complete the mappings between FMA classes and the different annotation terms [46,47]. This is particularly true for accommodating and reconciling different labeling schemes for the cerebral cortex. For example, the Talairach term Right Cerebrum.Frontal Lobe.Superior Frontal Gyrus.Gray Matter.Brodmann area 6 refers to an area in the gray matter of the right superior frontal gyrus that overlaps with Brodmann area 6. Whereas the gyrus is subdivided into regions based on topographical surface landmarks, Brodmann areas are regions defined on the basis of the underlying cytoarchitecture or cellular and laminar organization.

Although both types of regional partitions are in the FMA, neither Brodmann area 6 nor the gray matter of the right superior frontal gyrus had been partitioned to account for the overlap. We therefore reconciled both morphological and cytoarchitectural schemes into the FMA ontology with the following modeling pattern.

**Figure 2:**
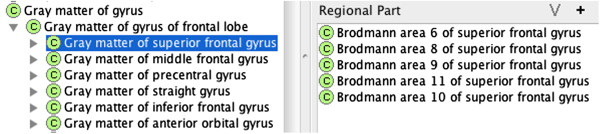
A listing of regional parts (i.e., Brodmann Areas) for the gray matter of the superior frontal gyrus.

**Figure 3:**
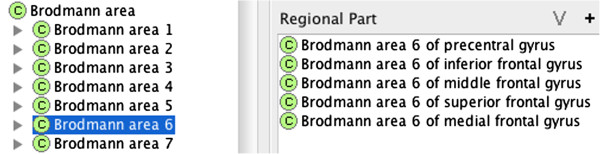
A listing of regional parts (i.e., cortical gyri) that intersect Brodmann Area 6.

First, we created a class for the Gray matter of the superior frontal gyrus, which overlaps with (i.e., ***has_regional_part***) Brodmann areas 6, 8, 9, 10 and 11 (Figure 2). Second, we created a class for Brodmann area 6 and model overlaps with the gray matter of the precentral, the superior frontal, the middle frontal, the inferior frontal and the medial frontal using ***has_regional_part*** relations (Figure 3).

**Figure 4:**
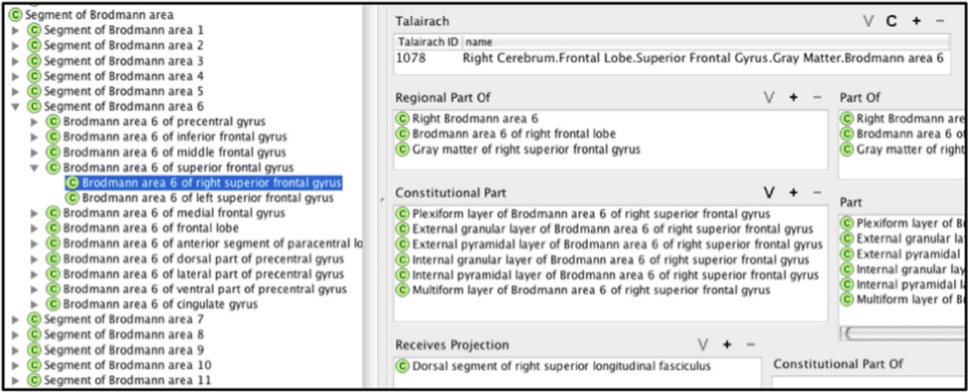
Part relationships of Brodmann area 6 of right superior frontal gyrus.

**Figure 5:**
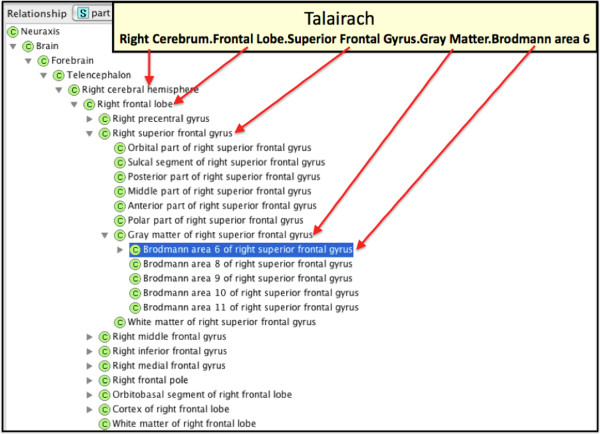
Correlation of Talairach label to part relationships of Brodmann area 6 of right superior frontal gyrus in the FMA.

Going back to our Talairach example above, we mapped it to the new FMA class called Brodmann area 6 of right superior frontal gyrus which ***is_a*** Segment of Brodmann area 6 and a ***regional_part_of*** both Right Brodmann area 6 and Gray matter of right superior frontal gyrus (Figure 4). And following the transitive ***part_of*** relation of Brodmann area 6 of right superior frontal gyrus up the FMA part hierarchy reveals that all the granularity levels implicitly encoded in the Talairach label are explicitly represented in the part hierarchy of the FMA (Figure 5). The latter is the kind of information that the ontology can provide to facilitate automated reasoning by any system.

#### ***Mapping Desikan-Killiany and AAL***

The labels used in FreeSurfer with the Desikan-Killiany (DK) atlas contain abbreviations and acronyms such as “Ctx”, “lh” and “wm” which mean “Cortex”, “Left hemisphere” and “White matter”, respectively. For example the term “ctx-lh-postcentral” maps to Gray matter of left postcentral gyrus. Many of the terms used in DK (e.g. ctx-rh-inferiortemporal) and AAL (e.g. Temporal_Inferior_Right) are customized and specific only to their respective projects. Therefore, some semantic interpretation is required to parse the meaning and interoperate with other atlas terminologies. In the FMA we provide a semantic framework that explicitly declares the intended meanings of the terms used.

We identified anatomical entities (i.e., classes) in the FMA that most closely correspond to a given brain atlas label. We then elaborated on the properties associated with each FMA class to provide additional relationships that capture information necessary to correlate with the labels from other brain atlases and NeuroLex (e.g., identifiers, preferred names, etc.). From the above examples, ctx-rh-superiorfrontal from DK is mapped to the FMA class Gray matter of right superior frontal gyrus and Frontal_superior_right from AAL to FMA class Right superior frontal gyrus.

**Figure 6:**
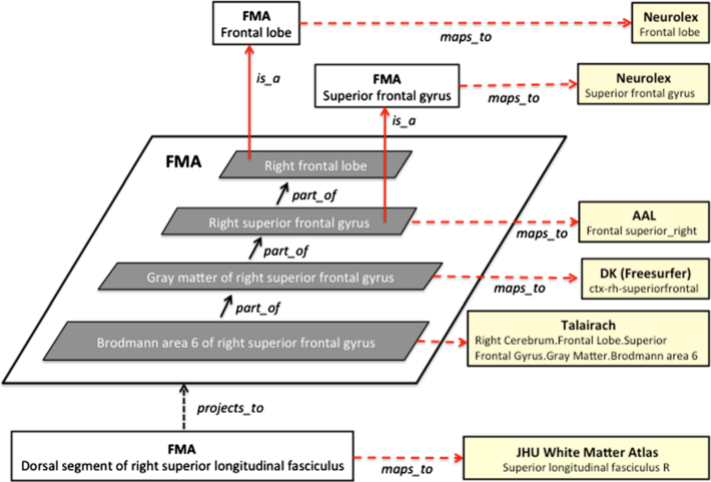
An example of how terms from brain atlases and vocabularies (right, yellow) can be correlated by mapping to the corresponding class in the FMA hierarchy.

Note that the structural entities represented by the different terms are at various levels of granularity, with Talairach, FreeSurfer and AAL at the levels of Brodmann area, gray matter of cortex, and gyrus, respectively. Furthermore a laterality attribute is specified for all three representations as opposed to NeuroLex, which does not require left/right attributes. However the FMA has the framework to correlate all the entities based on their ontological definitions and relationships as shown in Figure 6. In this example, the Talairach term is mapped to the FMA class Brodmann area 6 of right superior frontal gyrus, a ***part_of*** Gray matter of right superior frontal gyrus, the FMA class referenced by DK, which in turn is a ***part_of*** the AAL mapped entity Right superior frontal gyrus. The non-lateralized NeuroLex classes are then mapped via ***is_a*** relation to the lateralized entities represented in the other terminologies (e.g., Right superior frontal gyrus (AAL) ***is_a*** Superior frontal gyrus (NeuroLex)).

#### ***White matter and connectivity relationships***

There are a growing number of human neuroimaging techniques from the emerging field of connectomics [2,48,49] that can describe white matter connectivity at an increasing level of detail. Parallel ontological representations are required to capture and accommodate the newly derived or updated knowledge models these methods provide. This is necessary to establish precise and reliable structural-functional correspondence between disparate representations of white matter structures. Using new and classic neuroanatomical knowledge we have enhanced the FMA representation of white matter tracts and connectivity relationships between gray and white matter structures.

#### ***Partonomy of white matter structures***

**Figure 7:**
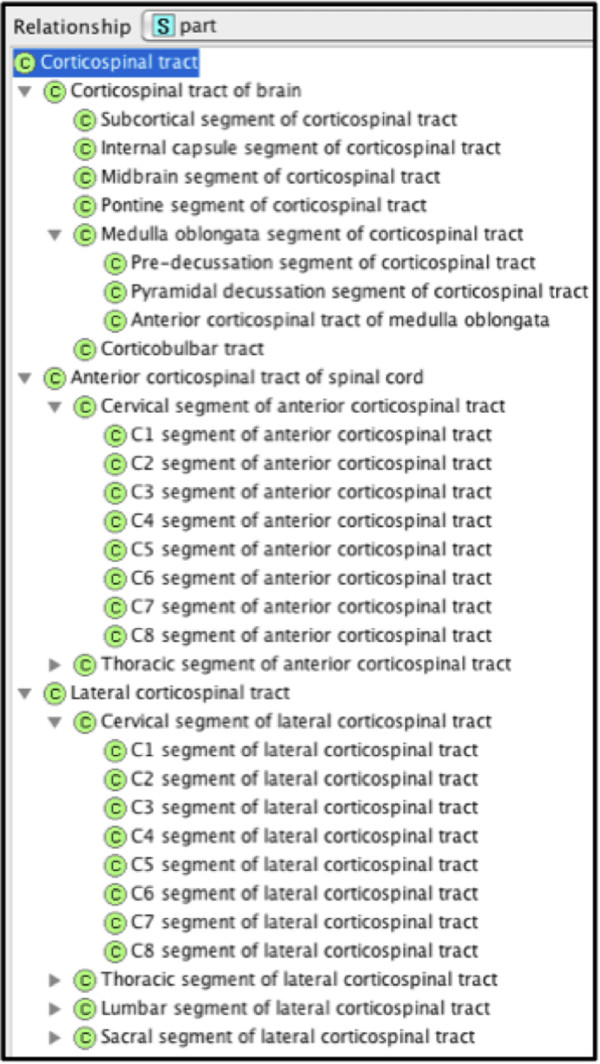
Regional partition of the corticospinal tract from the brain to the spinal cord.

We pursued a comprehensive spatio-structural representation of white matter tracts, particularly relating to partonomy and connectivity relationships. A good example addressed by this approach relates to the common practice of using the same term to represent both the entire tract and its segments, as in the case of the Corticospinal tract. In cases where only a very specific segment of the tract is to be identified, the indiscriminate use of a non-exclusive term for its annotation can lead to errors and inconsistencies, especially when machine-processing is involved. This can be avoided by properly declaring the parts of a structure with unique terms assigned to each part and only using the structure specific term for annotation. Figure 7 illustrates this approach using the Corticospinal tract (i.e., the complete structure) and all of its named segments/parts.

#### ***Granularity of connectivity relationships***

Connectivity between neuroanatomical entities entails relationships at different levels of granularity. Numerous terms have been used inconsistently to establish connectivity relationships between neurons, between nerve fibers or tracts and between gray matter structures. In this paper we proposed and gave definitions to specific connectivity types for different granular entities (Table 1).

**Table 1:** White matter connectivity terms and definitions

	
**Innervate**	A connectivity relation where a neurite of one neuron synapses with a neurite or a region of the soma of another neuron or a region of a muscle cell or a gland cell.
**Synapse_with**	A connectivity relation where there is apposition between the presynaptic membrane of a neurite of one neuron and the postsynaptic membrane of one or more neurites of another neuron or a region of a muscle cell or a gland cell and some form of neurotransmission is evident between them.
**Projects_to**	A connectivity relation where individual axons comprising a fiber tract originating from one or more brain regions synapse_with neurites or somas of a collection of neurons located in one or more other brain regions. This relation may be synonymous with ‘*terminate_in*’.
**Projects_from**	A connectivity relation where individual axons comprising a fiber tract are parts of a collection of neurons located in one or more brain regions. This relation may be synonymous with ‘*originate_in*’.
**Sends_output_to**	A subproperty of project_to relation where neurotransmission is sent from one brain region to one or more other brain regions.
**Receives_input_from**	A subproperty of project_from relation where neurotransmission is received by one brain region from one or more other brain regions.
**Has_pathway**	A connectivity relation where a collection of neurons located in brain region A sends_output_to a collection of neurons located in B via axons comprising the fiber tract from brain region.

**Figure 8:**
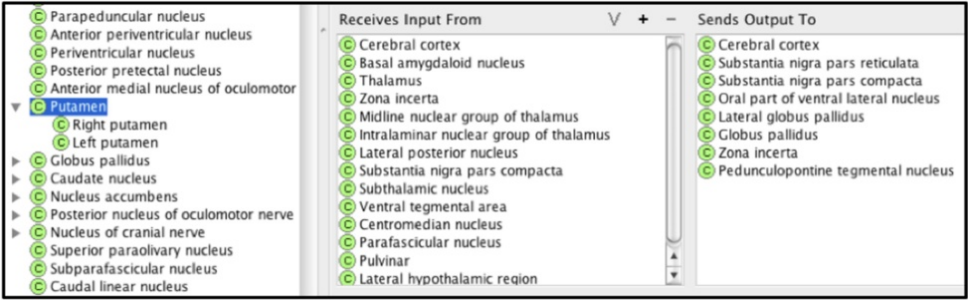
Connectivity relationships for the Putamen.

At the neuronal level, a neuron can ***synapse_with*** another neuron or a muscle fiber or a gland cell. Connectivity is at the subcellular level between the pre-synaptic membrane of a neurite of a neuron with a post-synaptic membrane of a neurite or soma of another neuron or with a region of a muscle fiber or a gland cell. White matter structures at the nerve or tract-level (i.e., collection of axons) ***projects_to*** and ***projects_from*** any region of the neuraxis (i.e., a term referring to both brain and spinal cord). For example, the Dorsal segment of superior longitudinal fasciculus (i.e., SLF I) ***projects_from*** Brodmann area 6 of superior frontal gyrus and ***projects_to*** Brodmann area 5 of superior parietal lobule. Finally, we created ternary relationship types that model neural connectivity between any two regions of the neuroaxis connected by white matter. Gray matter structures ***receives_input_from*** and ***sends_output_to*** other gray matter structures, as shown in Figure 8 for Putamen.

The connectivity relationships we propose capture the essential levels needed to express how information is communicated throughout the brain. However, several other efforts are working on the issue of neural connectivity relations. For example, the OBO Relation Ontology [50] proposes relations such as ***has_fasciculating_neuron_projection*** and ***axon_synapses_in***. As additional relationships are defined, the FMA will provide a framework to accommodate these terms for further refinement of connectivity representation.

#### Cerebral sulci

**Figure 9:**
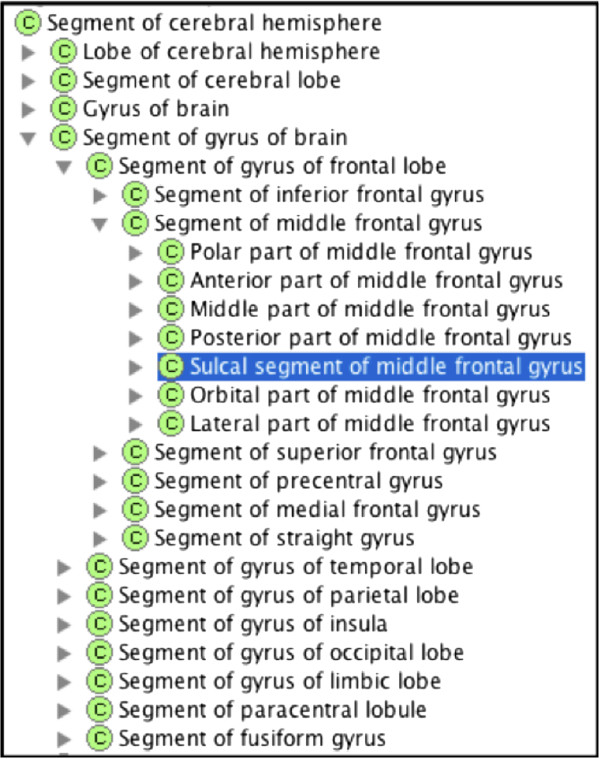
**Regional parts of the middle frontal gyrus.** Sulcal segment of middle frontal gyrus is the part located in the sulci.

Among immaterial anatomical entities, particular attention was directed to anatomical spaces such as the cerebral sulci. Sulci are defined in different contexts, depending on the operational needs of the users. In some labeling protocols, sulci are treated as 1-D lines that serve as boundaries of gyri, whereas in surface-based parcellation models they are spaces or grooves that surround the gyri. The PALS-B12 atlas from Caret [51], a significant labeling scheme of widespread utility, involves the use of sulci to identify and contour “buried” cortex among gyri. Here the term “sulcus” denotes a 3D volume, the segments of gyri located in the furrows. We disambiguated the representation of sulcus by treating it as an anatomical space and for the area of the gyrus in the sulcus, we regarded it as an anatomical structure that is part of the gyrus and classified it as sulcal segment of a gyrus under Segment of gyrus of brain. As shown in Figure 9, the middle frontal gyrus consists of several regional parts or segments, one of which is Sulcal segment of middle frontal gyrus and the rest are parts of the gyrus that are externally visible. With this approach, the entire middle frontal sulcus can be modeled as belonging to two gyri – both the Sulcal segment of middle frontal gyrus and Sulcal segment of inferior frontal gyrus.

### Utility and discussion

Extensions to the neuroanatomical axis of the FMA were motivated by use cases in ontology development, knowledge retrieval, and data integration. In this section we describe several use cases for our work and discuss how reusable and computable anatomical knowledge captured in the ontology can be utilized to solve real-world problems.

### Neuroanatomical knowledge reuse

The FMA is a reference ontology that can be imported into other ontologies as a way to reuse curated knowledge about anatomy [37]. RadLex [40] is an ontology composed of standard terms for the domain of radiology, including imaging observations, characteristics, and techniques, as well as diseases, radiology reporting terminology, and anatomy. For anatomy, RadLex incorporates a subset of the FMA that is relevant to the radiological scale of analysis [41].

RadLex also contains knowledge beyond anatomy that enables additional radiology oriented use cases such as human brain imaging. For example, a digital brain label can indicate the anatomical structure that a set of image coordinates pertains to in a brain template, whereas RadLex can be used to describe key aspects of a neurological imaging examination including modality, technique, visual features, anatomy, findings, and pathology. By incorporating neuroanatomical content from the FMA, RadLex enables rich dataset annotations and provides a means to correlate and integrate the findings with other external data and studies as discussed in the following section.

### Ontological knowledge retrieval

To leverage the knowledge we encoded into the FMA the ontology can be accessed using a query engine. For this purpose we used the Query Integrator (QI) as an underlying technology [52] to query the neuroanatomical content of the FMA as represented in OWL-Full. The QI is a Web-based query management and execution system that enables queries over any Web-accessible data or knowledge source (e.g. ontology). The QI supports multiple query languages, including SPARQL [53] for RDF [54] data sources. QI queries may be stored for reuse, executed via RESTful Web services, and chained together to form query pipelines. This latter capability allows the results of ontology queries to be joined with data queries to answer more interesting questions than are possible based on the data alone.

#### ***Dataset annotation and “intelligent” query***

As reported in Turner et al. [47], the FMA was used to annotate a large dataset of task-based functional MRI (fMRI) signal activations in subjects with schizophrenia and healthy subjects. The activation locations were annotated with neuroanatomical labels from the Talairach Daemon [28,55]. These labels combined cytoarchitectural labels from one method for labeling brain regions, with morphological terms based on sulci and gyri. The FMA was extended to include intersections between label pairs when regions overlap. For example, within areas covered by the label Inferior temporal gyrus exist areas covered by the label Brodmann area 20. Therefore, ***part_of*** the Inferior temporal gyrus is ***part_of*** Brodmann area 20, and ***part_of*** the Inferior temporal gyrus is not in Brodmann area 20. Conversely, Brodmann area 20 has parts that are in the Inferior temporal gyrus and parts of Brodmann area 20 which are in other gyri. This extension of the ontology in conjunction with a reasoning engine allowed novel questions to be asked about the data.

#### ***3D anatomical model management***

**Figure 10:**
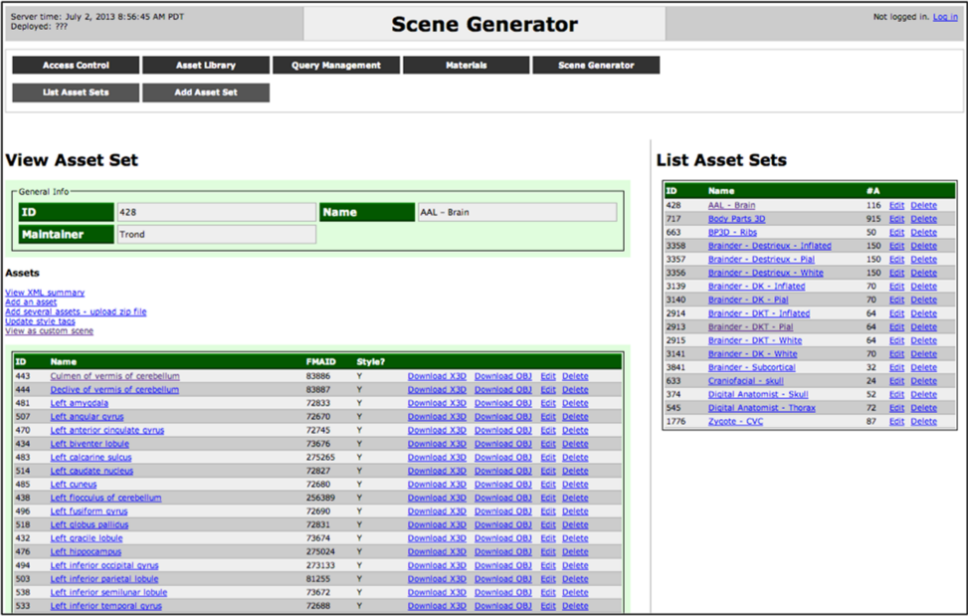
**Screenshot of the 3D model asset manager component of the scene generator displaying a list of asset sets on the right and the selected asset set on the left (AAL - Brain).** Scenes are generated from the selected asset set based on queries to the FMA.

In biomedical education and research, 3D surface models are useful and commonplace. For example, a researcher using the brain atlases described above may generate neuroanatomical surface models from a patient’s MRI, where each model is a different brain region. While these models can be organized using naming conventions or directory structures, it may be more meaningful to annotate models using terms from the FMA. Similar to our work on annotating tabular datasets, the knowledge in the FMA can be queried and used to reason about which 3D models to select and display in a 3D scene. We have developed a prototype scene generation system that implements this idea and will allow users to create Web-based 3D scenes using the results of queries over the FMA or data sets annotated with FMA identifiers (Figure 10).

**Figure 11:**
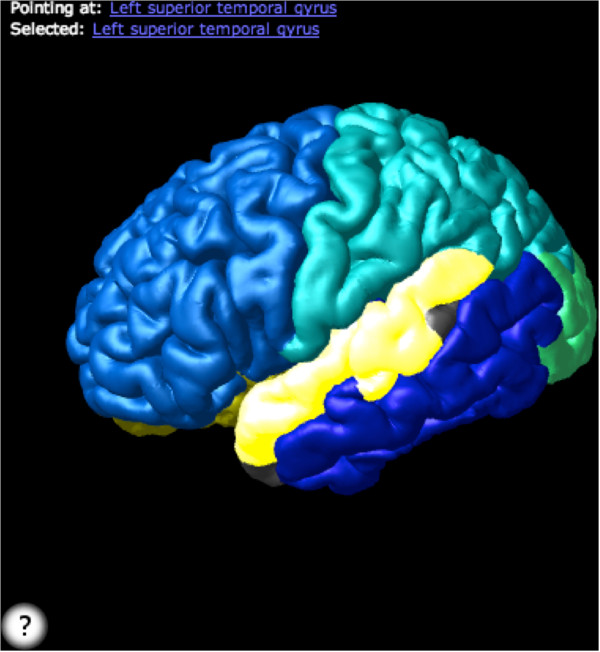
**A view of the DK left hemisphere parcellation with the left precuneus selected.** A link to this scene can be found at: http://purl.org/sig/docs/neurofma-jbs.

**Figure 12:**
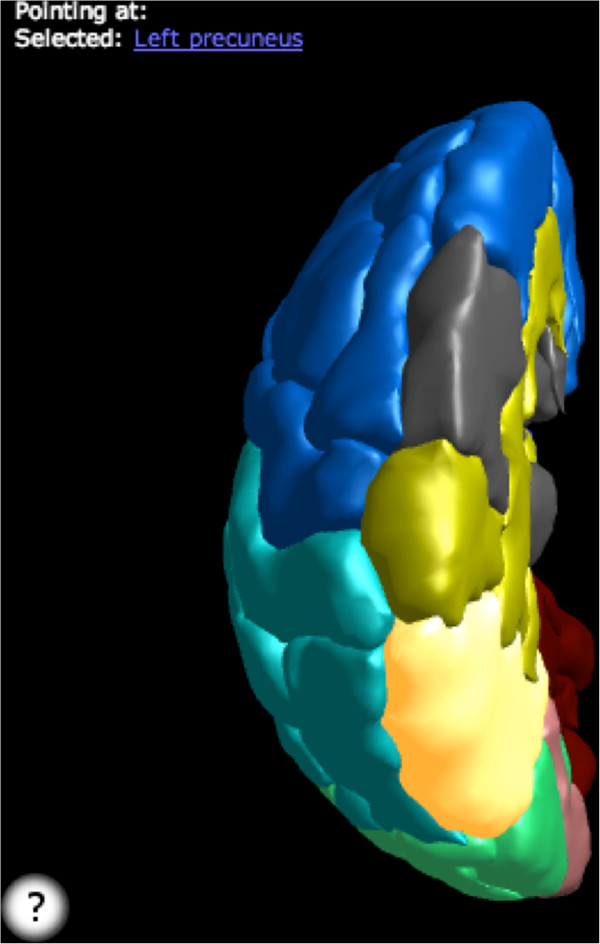
**A view of the AAL left hemisphere labeling with the left superior temporal gyrus selected.** A link to this scene can be found at: http://purl.org/sig/docs/neurofma-jbs.

The system provides access to several biomedical 3D model sets, including models generated from the brain atlases described above (e.g., AAL and DK) and allows users to upload their own models. Scenes can be constructed by hand or using queries, and queries can be shared between users and customized using parameters. Since all scenes are rendered using WebGL, they can easily be embedded within any website or Web-based publication. For example, users of this system can access knowledge in the FMA to generate a scene showing all portions of the brain with blood supply from the middle cerebral artery or all structures connected by a given white matter tract. Similarly, scenes can be generated to display different model sets of the same structures such as 3D models of the left hemisphere in the DK (Figure 11) and AAL anatomical labeling schemes (Figure 12).

This tool currently provides users with the ability to manage 3D model sets, query annotated models, and visualize query results as 3D scenes. As this tool matures it will also incorporate support for building scenes from connectivity relationships, visualizing the correlation between model sets, and displaying volumetric anatomical images. In addition, our tool provides model management features that facilitate sharing and reuse of model sets. These tools are also useful in validating and exploring the FMA and other terminologies, as well as model sets based on these. Misalignments, missing structures, and issues with coordinate sets readily become apparent in query-driven scenes and, in many cases, can be remedied from within the tool and re-exported for use elsewhere. Similarly, visualizations can be composed that relate the structures defined in different terminologies to one another. As mentioned, this system is currently under development; we anticipate publishing and releasing it to the community in the near future. Links to any publications associated with this tool will be available on the supplementary materials page (http://purl.org/sig/docs/neurofma-jbs).

## Conclusions

We demonstrated that the framework provided by the FMA ontology can be extended to accommodate and correlate the terms used in three human brain labeling schemes and NeuroLex. We then used the enhanced FMA to highlight use-cases for neuroanatomical knowledge reuse and retrieval. The FMA was found to sufficiently capture and clarify the relationships between different anatomical labeling schemes necessary to fulfill the use cases.

As a result, the disciplined and principled approach in the FMA lays the foundation for:

1. An ontology-based standard for anatomical data annotation

2. Queries that can use the ontology to infer anatomical relationships in data

3. Data visualization systems that incorporate anatomical knowledge

4. A “meta-atlas” that harmonizes different brain labeling protocols

5. A unifying anatomical framework for integrating a variety of biomedical data

While this effort advances state-of-the-art knowledge representations of human neuroanatomy, further work is needed to address the brain atlas concordance problem. Symbolic representations of anatomical labeling schemes alone are not sufficient to model the spatial information in brain atlases. Computational frameworks, such as proposed by Bohland, et al. [25], provide quantitative measures of spatial concordance but do not address the issue of lexical mappings. A hybrid framework that integrates quantitative information with ontologies would offer a more comprehensive solution to reconciling neuroanatomical labeling schemes. Additionally, the purely structural approach taken by the FMA only accommodates anatomical descriptors, and the need for functional divisions of the brain calls for future development of a “functional brain labeling ontology”.

## Availability and requirements

Latest Release:

Foundational Model Explorer (Online):

http://sig.biostr.washington.edu/projects/fm/FME

Frames:

http://sig.biostr.washington.edu/projects/fma/release/index.html

OWL-Full:

http://sig.biostr.washington.edu/share/downloads/fma/FMA_Release/alt/v3.2.1/owl_file/fma_3.2.1_owl_file.zip

Licensing:

Creative Commons Attribution 3.0: http://creativecommons.org/licenses/by/3.0

## Abbreviations

AAL: Automated anatomical labeling; FMA: Foundational model of anatomy; fMRI: Functional magnetic resonance imaging; MRI: Magnetic resonance imaging; NIF: Neuroscience information framework; NIFSTD: Neuroscience information framework standard ontology; OBO: Open biomedical ontologies; QI: Query integrator; RO: Relations ontology; ROI: Region of interest; TD: Talairach daemon.

## Competing interests

The authors report no competing interests with the work described in this manuscript.

## Authors’ contributions

BNN provided expertise on brain atlases, extracted connectivity information from the literature, and coordinated use case efforts. JLVM is a primary author of the FMA and manually incorporated terms into the FMA using Protégé. TTN is the primary developer of the model management and visualization application. MEM provided expertise on NIF, NeuroLex, and neuroanatomy. JAT provided expertise on neuroanatomical dataset annotation and brain imaging. DLR provided expertise on RadLex. JFB oversaw all research activities and provided expertise on the FMA and QI application. All authors reviewed, edited, and approved of the manuscript.
